# A Principled Approach to the Origin Problem

**DOI:** 10.1007/s11084-015-9444-3

**Published:** 2015-07-16

**Authors:** Masashi Aono, Norio Kitadai, Yoshi Oono

**Affiliations:** Earth-Life Science Institute, Tokyo Institute of Technology, 2-12-1 Ookayama, Meguro-ku, Tokyo 152-8550 Japan; PRESTO, Japan Science and Technology Agency, 4-1-8 Honcho, Kawaguchi-shi, Saitama, 332-0012 Japan; Department of Physics and IGB, University of Illinois at Urbana-Champaign, 1110 W. Green Street, Urbana, IL 61801-3080 USA

**Keywords:** Complex systems, Prebiotic chemistry, Protometabolism

## Abstract

The key issue of the origin of life is the origin of a complex system rather than the abiotic formation of various organic substances, small and large. To consider this “origin problem” it is advantageous to abstract some principles from biology and statistical physics to guide our approach. Referring to these principles, we aim to construct a chemical system called “protometabolism,” which would be a precursor of metabolism.

## Introduction

What is the cultural significance of the study of the origin of life? Physics as an empirical science depends on observations by us, macroeukaryotes. On the other hand, physicists wish to deduce everything we can observe from physics. Thus, a naturalistic *Weltanschauung* demands consistent understanding of physics and biology. Consequently, the origin study is its key ingredient.

The origin of life has been studied for more than a century; it is more than 50 years since Miller’s (1953) epoch-making experimental study. During this time Wächtershäuser’s models (1990) respecting general principles of chemistry and logic (and encouraged by Popper) may look rather peculiar, but it is important to be explicit about basic modes of our thinking. In this article we try to be as explicit as possible about the principles that regulate our approach to this fundamental question^1^; we refer to our approach based on basic rules as a “principled approach.”

## Principles

### Characterizing Life?

There are many attempts to define life and the so-called NASA definition (Joyce 1994; Luisi 1998) seems the most popular; it says a living system (or an organism) is a self-sustained chemical system capable of undergoing Darwinian evolution. Is life a property of individual organisms or that of a population? When we ask whether a person is alive, we never care whether she can reproduce and whether her offspring can evolve. Collective features such as evolvability, heredity, and variation should be deducible from the definition of life (if any).

We are, however, more modest than the predecessors, so we will not try to define life. Instead, we will try to find a larger set (i.e., a supset) which includes living things that is not as hard as life itself to characterize and that excludes all obvious non-organisms like computers. Everyone agrees that organisms are complex systems, and that life is a complex phenomenon, although no clear understanding of the word “complex” seems agreed upon. Therefore, we wish to try to characterize the supset above as the set of complex systems/objects. To this end we need a critical analysis of the concept “complexity” (Oono 1998, 2012): Complex systems (CS) are systems requiring highly nontrivial auxiliary conditions (e.g., initial and boundary conditions) to emerge, called “fundamental requisites (FR),^2^” which cannot be prepared readily without devoting a great deal of resources in terms of time and information. All organisms descend from other organisms as Pasteur demonstrated; we have our parents. This “Pasteur’s principle” holds, because humans never self-organize (spontaneously emerge) within a short time without our parents who provide FR. Thus, FR and CS constructed from FR may be characterized as:

I. [Ontogeny] Without FR no CS emerges within a short time scale ${\tau}_O$. In other words, ontogeny of CS requires FR.
II. [Pasteur’s principle] FR emerges only from CS. (Since we know there was no CS long ago, Pasteur’s principle cannot always be correct.) To make FR spontaneously without CS, a long time scale, ${\tau}_P$, is needed (${\tau}_P/{\tau}_O\gg 1$).
III. [Harsh world] Both CS and FR are vulnerable to noise and never survive beyond the time scale ${\tau}_S$ defined as ${\tau}_O\le {\tau}_S\ll {\tau}_P$. In particular, “interconversion” FR ↔ CS becomes impossible after ${\tau}_S$.

III + I implies CS (eventually, but on the time scale ${\tau}_O$) dies (i.e., is destroyed without possibility of resurrection). Thus, *memento mori*, if you are interested in complex systems. That is, for example, how cell dies is an important research topic of biology as a study of complex systems. Intuitively, FR is less vulnerable to noise than CS, but cannot multiply in contrast to CS. Incidentally, notice that viruses are CS.

### Evolution is Implied

If we may assume that a CS exists for a very long time ($\sim {\tau}_P$), the FR-CS cycle (generation) should have repeated numerous times. Since FR is corrupted by noise, there must be a mechanism to keep FR viable. Even “copy-errored” FR can be preserved, because most small modifications of FR should not be very harmful. Thus, the only way to keep FR viable for many generations (i.e., to produce a CS that can produce the next generation of FR) is to select a “good” one among “corrupted” FRs. Since comparison of information (e.g., sequences) is a computationally hard problem,^3^ no syntactic comparison is practical. Besides, when compared, it is impossible to tell which is not corrupted. Thus, the only practical comparison is through competition of the resultant CSs. That is, the Darwinian process is required to maintain FR. Notice that even if the original FR were intelligently designed, as long as there have been no divine interventions (i.e., miracles) ever since, the only way to maintain a viable FR is through Darwinism.

### Origin and Metabolism

The origin of FR is a key question in the origin problem. The production of FR requires resources that cannot be acquired by FR themselves. Thus, a CS that can somehow obtain needed resources intervenes in the reproduction of FR (FR → FR). During the resource accumulation period during which a CS becomes mature, the repairing of damages in CS is required. Recall that the main energetic cost in the bacterial life process is amino acid and protein synthesis, consuming $\sim 3/4$ of the cell’s ATP budget, with RNA monomer and polymer synthesis consuming only about 10 % (Stouthamer 1978). The free energy expenditure is basically to battle (or for battling) against noise (against damaged proteins which cause proteotoxicity).

A CS is needed to harness free energy through metabolism, but the Pasteur chain FR → $\cdots$ → FR requires preservation of “genetic” information. Life requires both. The origin of genetics is a hard problem, so let us first concentrate on the origin of metabolism, although it is only a small portion of the origin problem.

### Origin = Origin of a System

The origin of life is not a question about the abiotic synthesis of biomolecules, but rather about a system, which includes a precursor of the metabolic system. We will never be able to make life from an organism-free soup available by sonicating a culture solution full of *E. coli*, that surely contains all the building blocks of life.

To construct a plausible scenario for the origin of metabolism, we first look at extant metabolic pathways and distill a few “principles” we believe must be obeyed. Every metabolic pathway must be controllable. Most metabolic pathways are kinetically hard without enzymes. No spontaneous reactions as unruly as the HCN polymerization appear. This suggests that even prebiotically the precursor reactions do not proceed without catalysts, and that no spontaneously feasible reactions are crucial. The lack of spontaneity may be a key feature of biological systems. Even at the earliest stages of biological evolution, and possibly before, spontaneity should have been restricted.

Reactions with high activation energy barriers, are potentially regulated and controlled more easily, so they can be exploited to make organized systems. On the other hand, very spontaneous reactions are too uncontrollable to be used as a part of an organization, if they could stand by themselves without help from enzymes.^4^ We summarize these observations as the “principle of narrow gates”, referring to the following prophetic passage: *Because straight is the gate*, *and narrow is the way*, *which leadeth unto life*, *and few there be that find it* (Matt. 7 [14]).

Since enzymes usually preserve the purely organic-chemistry reaction mechanisms,^5^ and since the evolution of metabolism is largely dependent on the evolution of enzymes, the above idea is generally supported also by the so-called “chemical continuity” between prebiotic and metabolic reactions that has been utilized by many predecessors in the field.

### Chemical “Qualification”

A chemical network is a set of chemicals that are connected with each other via reactions (or reaction chains) and these reactions actually proceed (that is, they are not simply feasible). Suppose we add a chemical that is *not* connected to the network beforehand. If the added chemical and any component of the network do not react, the added chemical can only be a part of the environment of the network.^6^ On the other hand, there are two possibilities, if the added chemical and the network component(s) do react. Since the added chemical is not a product of the network, if it is not amply supplied, then the reaction with the network component(s) consumes it. Only when the added chemical is steadily supplied or a part of another running network, it can be qualified to be incorporated into the joined network. Thus, unless the unconnected chemical is amply supplied, it is irrelevant to the network (except perhaps as a cofactor). This should be called the principle of “chemical qualification,” so to speak. In other words, the compounds not intrinsic to a given chemical network are irrelevant to it. Thus, for example, the studies of organic compounds in meteorites and the interstellar medium, etc., could only contribute to issues that are related to solvents and cofactors. Combinatorial chemistry would give only a limited contribution to the origin problem, since most of the unsynthesizable chemicals appearing in the combinatorial efforts, if reactive, will be wiped out quickly by the network.

### Hints from Metabolism

What sort of chemical system should we try to construct? Let us look at the metabolic networks of prokaryotes more closely. We will discuss three important observations: (i) the existence of a rather universal carbon anabolic hub, (ii) non-primordiality of methanogens, and (iii) impossibility of utilizing small molecules (N_2_, H_2_, CO_2_, etc.). The most important observation is, as stressed by Fuchs (2011), that there are not so many ways to assimilate carbon. Although reverse TCA (rTCA) has been singled out as the original carbon anabolic pathway by some authors (e.g., Smith and Morowitz 2004), the supporting arguments are slim. We must also take Orgel’s criticism (2008) very seriously that the primordial realization of the rTCA without efficient enzymes was prohibitive. Furthermore, excessive emphasis on the importance of autocatalysis may be unwise. Our contention is that the fountain of life mentioned below allows us to do without cycles; cycles may well be later inventions.

If one wishes to construct a fully autotrophic scenario, one should start with C1 compounds (henceforth, C*N* compound implies that the compound has *N* carbon atoms) as Russel, Taylor and others (e.g., Martin and Russell 2007; Nitschke and Russell 2013) have been attempting. They paid very close attention to methanogens, but are methanogens really primordial? In most instances, compounds which serve as substrates for methanogens are produced as end products of various bacterial and eukaryotic fermentations and anaerobic oxidations, so methanogens do not gain a rich living (Ferry 2004). Thus, methanogens are likely “high-tech” organisms evolving only after lucrative resources were exhausted. Complicated cofactors and often required chemiosmotic phosphorylation also argue against their primordiality.

The third important observation may be that enzymes handling small molecules (such as N_2_, H_2_, CO_2_, etc.) are all sophisticated complex enzymes,^7^ so it is natural to conclude that initially (pre)biological systems could not utilize inorganic small molecules such as N_2_, H_2_, CO_2_, etc. Thus, the origin of life must have required more reduced and/or larger compounds such as NH_3_, CO, acetate, etc.

### Need for Nonequilibrium Conditions

If we prepare a sufficiently concentrated “(pre)biosoup” and keep it warm for 1 Ga in a closed bathtub, what will happen?^8^ Thermodynamics tells us that we will have an equilibrium mixture and nothing remarkable will happen. The preparation of biomaterials is not a key issue for the origin of life.

As Bénard convection illustrates, an auxiliary condition that maintains the system only slightly out of equilibrium drastically changes underlying probability assignment to elementary microstates. Thus, some convenient nonequilibrium boundary conditions must be imposed externally for the origin of life. Since the origin of life is an origin of chemical system, free energy and raw chemical materials must be supplied. Then, the most natural nonequilibrium condition is a sort of “fountain” of high energy compounds. This type of the boundary condition is what we call the “fountain of life.” It can be a fountain of a single or a few compounds (say, acetate alone).

Incidentally, in emphasizing disequilibrium, some seem to conclude that irreversible thermodynamics is relevant to the origin problem, but this is misleading.^9^ In contrast, equilibrium thermodynamics is very useful in metabolism. Cells are largely in compartmentalized equilibrium states in which compartments (which may be distinct degrees of freedom and need not be spatial compartments) are very close to equilibrium themselves. A key point of equilibrium thermodynamics is that for a compartmentalized system 1 + 2

$$ \varDelta {S}_1+\varDelta {S}_2>0\kern0.28em \mathrm{with}\kern0.28em \varDelta {S}_1<0\kern0.28em \mathrm{and}\kern1mm \varDelta {S}_2>0 $$

is possible. If $\left|\varDelta {S}_1\right|$ is large, so must be $\varDelta {S}_2$. A fountain of life must conveniently furnish such a system as system 2.

If there is no supply of high-chemical-potential materials, nothing would happen, even if all the building blocks are somehow amply supplied (recall the biosoup made by sonicating *E. coli* culture solution). One might say light (UV), electric discharges (thunder), cosmic rays, etc., could supply sufficient high energy, but unless these high energy sources are organized (or harnessed) as chemical free energy, we cannot use them to construct a chemically organized system.

### Organizing Center

**Fig. 1:**
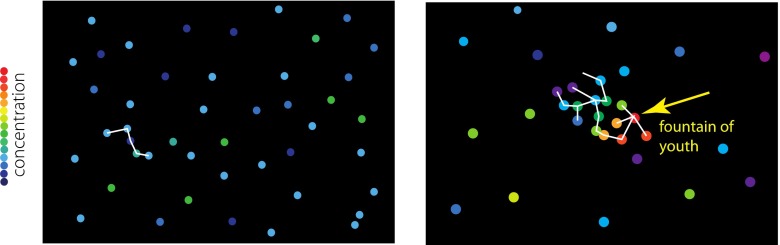
*Left*: A chemical space without any “organizing center.” *Colored dots* correspond to various organic compounds whose concentrations are color-coded. All concentrations are low. *Right*: A chemical network connected with an organizing centery

The construction of a chemical system may be illustrated with the aid of a chemical space in which all the chemical compounds are arranged according to the chemical closeness, as shown in Fig. 1. The chemical closeness between the compounds may be measured by the number of elementary reactions (i.e., unit reactions such as hydration/dehydration) that connect with each other.

The so-called heterotrophic scenarios (cf. Lazcano and Miller 1999) assume that network chunks, denoted by white edges in Fig. 1 (Left), are spontaneously formed in a “warm little pond.” However, according to our principles of narrow gates and chemical qualification, for that chunk to be recognized as a part of a pathway, it must be “operated”; it is not a mere chemical possibility, but should actually occur steadily. To this end, the starting material of the pathway must be amply supplied. That is, the chunk must be connected to an organizing center or the fountain of life; even within the heterotrophic scenario an organizing center is needed to make a system. The important distinction of the origin scenario does not lie whether it is the so-called heterotrophic or autotrophic scenario, but whether there is an organizing center or not.

If we have an organizing center, then we may have Fig. 1 (Right). Here, compounds unrelated to the fountain are also depicted as on the left hand side of Fig. 1. These unconnected compounds do not participate in the network supporting protometabolism^10^; they might act as catalysts (and cofactors) or solvent components, but they are never reactants.

A chemical network spontaneously emerges only if edges are “feasible,” that is, only if elementary chemical reactions corresponding to the edges actually occur. The reactions denoted by the white edges may have low yields, so eventually the network dwindles near the periphery. Notice that the chemical system around the fountain is just like a flame around a burner. This comparison is rather apt, since life has a lot of common features with fire. However, there is a crucial difference between the reactions in the flame and in the organism. Most reactions in the organism do not proceed spontaneously without enzyme catalysis, as it can be confirmed in metabolic tables or biochemistry metabolic charts (e.g., Voet and Voet 2011). An organism is, even chemically, a compartmentalized metastable system, as mentioned previously. In flames, the reactions are spontaneous chain reactions (mostly radical reactions) without any hope of control as unruly as the HCN polymerization system. Various fountains could be imagined in the surface environments on the primordial continents of the Hadean Earth, as suggested by Maruyama (Dohm and Maruyama 2014); the candidates would be locally reduced environments such as serpentine-hosted hydrothermal systems in caldera lakes (Maruyama et al. 2013).

## Strategy

Our starting point should be C2 compounds, possibly acetate and related compounds (instead of CO_2_, CO or formate is the C1 carbon source, if we wish to start with C1 compounds as can be seen from reaction difficulties inferable from the prokaryote biochemistry). Spontaneous unruly reactions are avoided, and only those reactions that require catalysts are sought, following the principle of the narrow gates; that is, kinetically hard reactions with large $-\varDelta G$. Although we do not aim at constructing the rTCA, some representative compounds such as pyruvate, oxaloacetate, succinate and $\alpha$-ketoglutarate are considered in our project.

The rTCA may have come much later, maybe even after the TCA itself; it could well be a secondary streamlined version of the carbon assimilation pathway, so we have no intention to realize rTCA itself prebiotically. Synthesis of amino acids, and other metabolites are the second stage of our study.

Our principled approach is based on the scenario that requires an organizing center(s), i.e., (some sort of) the fountain(s) of life.^11^ Also we follow the general idea of chemical continuity that has been used by many predecessors in this field (e.g., Lazcano and Miller 1999). This could imply several “principles.” The principle of the narrow gate already mentioned is a typical one. Another important principle is to avoid the classes of “beneficial”^12^ or “high tech”^13^ reactions not being used in extant biological systems. A “beneficial” class of reactions is likely to remain at least as relicts in some extant organisms,^14^ perhaps adapted to special niches. Such organisms may not have yet been discovered, but this possibility is becoming increasingly slim due to the progress of metagenomics. According to this principle, the use of materials such as semiconductor particles (Eggins et al. 1998; Sadeghi and Nejat 2013; Tvrdy et al. 2011), which are not found in extant organisms, would not be plausible.

A very serious consequence of the chemical continuity is that there could not be the “pure” RNA world (cf. Carter et al. 2014) requiring metabolic ribozymes. All the ribozymes in actual organisms are all involved in the reactions concerning nucleic acids (particular examples are ribosomes and splisozomes). In particular, there is no ribozyme relevant to metabolism per se (especially, carbon metabolism). Thus, we can safely assume that there was no metabolic ribozyme. Since an RNA world would still require a metabolism to process raw materials, this suggests that there was no pure RNA world, but protein enzymes must have existed when polynucleotides existed.

The principle of chemical qualification implies that if no polymerization mechanism is invented as a part of the evolving chemical network, polymers are useless as reactants.^15^ Perhaps polymers might have been produced abiotically, but the resultant polymers are, especially if stable, nothing but organic compounds just as woody materials of the Carboniferous without lignicolous fungi (Floudas et al. 2012).

The concept of chemical continuity is sometimes criticized, because protometabolism might have been much closer to geochemistry than to present-day metabolism (cf. Lazcano and Miller 1999). However, at least the very core part is almost universal (i.e., not many possibilities exist). There must be chances that the compounds appearing in the extant metabolism and those in protometabolism are identical, so the reactions are related.

Even if one assumes some sort of takeover, there must be its remnants somewhere.^16^ Therefore, we dismiss most takeover stories, and thus chemical continuity implies the continuity within organic chemistry.

It might be possible to approach the origin problem more theoretically. If we study life at an elementary time scale not much shorter or longer than 1 ms, and near temperatures at which water is liquid, as long as polymers are crucial, then life must be carbon based. Therefore, the relevant chemistry is organic chemistry and how to make materials for polymers is a fundamental issue we must answer.

## Scenario

### Our Scenario, its Beginning Portion

If the system is fully autotrophic, it should start from CO_2_, or at least from some C1 compound. Indeed, Huber and Wächtershäuser (1997) synthesized acetate from methanethiol and CO in the presence of metal sulfide, although its actual relevance to the origin problem may be questioned. However, there is also good reason to believe that acetate was available on the primitive Earth, because the atmosphere contained CO in the Hadean.^17^ Thus, we start from acetate. Incorporating CO_2_ into C2 compounds (in particular, acetate) is thermodynamically very unfavorable, so some special means is needed. In extant anabolic reactions, ATP is used to overcome thermodynamic hill climbing. However, ATP is quite inert without appropriate enzymes, so more reactive high energy compounds are needed. To this end Gale’s observation (1951) is relevant: the hydrolysis of pyruvate

$$ {\mathrm{CH}}_3\mathrm{COCOOH} + {\mathrm{H}}_2\mathrm{O}\ \to\ {\mathrm{CH}}_3\mathrm{COOH} + \mathrm{HCOOH} $$

requires the presence of acetyl phosphate as an intermediate:

$$ {\mathrm{CH}}_3\mathrm{COCOOH} + {\mathrm{H}}_3{\mathrm{PO}}_4\to\ {\mathrm{CH}}_3{\mathrm{COOPO}}_3{\mathrm{H}}_2 + \mathrm{HCOOH}. $$

Thus, if we have supply of acetyl phosphate, pyruvate may be obtained via reaction with CO or formate, since we may shift the equilibrium of the above reaction to the pyruvate side. The question is how to obtain acetyl phosphate.

Several mechanisms have been proposed for the abiotic synthesis of acetyl phosphate on the Hadean Earth, including UV-driven phosphorylation of thioacetate (CH_3_COSH + H_3_SO_4_ → CH_3_COOPO_3_H_2_ + H_2_S) in the presence of uracil as a catalyst (Hagan 2010) and a radical reaction between acetate and phosphite radical (•PO_3_^2−^) formed from the corrosion of schreibersite (Fe_3_P) in aqueous solution (Pasek et al. 2007).

If the pyruvate problem is solved, then the next question is naturally the C4 compound. No one has synthesized oxaloacetate from pyruvate under reasonable geochemical conditions. This is again very likely due to the steep thermodynamic uphill reaction of adding one more carbon to C3. If we look at the formation of oxaloacetate from pyruvate in extant organisms, CO_2_ is activated with cofactors such as thiamine pyrophosphate or biotin. These cofactors are unlikely to be available initially.

Another approach used is the path via phosphoenol pyruvate (PEP), but this compound does not look easy to synthesize prebiotically without ATP. One idea to circumvent this difficulty is to use borate, which has been shown to stabilize certain enols as exploited by Benner et al. (2012). It may even be possible to realize gluconeogenesis almost geochemically with the aid of borate, because at least thermodynamically the hardest ascending hill is overcome with the aid of borates. A more complete picture of a possible protometabolism will be given a fuller exposition elsewhere.

We wish to add one important consequence of protometabolism. Chemical continuity logically implies that precursors of metabolism before the origin of life must not be distinguished clearly from biotic reactions through isotope studies, because the origin of protometabolism and even fairly complete carbon anabolisms with enzyme precursors of oligopeptides must have been far before the origin of life; only when the origin of life and the origin of metabolism may be identified can we conclude that isotope studies are meaningful.

### How to Improve Protometabolism?

We have briefly discussed the very beginning portion of protometabolism and next discuss how a polymerless protometabolism could evolve to a full-fledged metabolism. If protometabolic pathways can be realized geochemically and show chemical continuity with modern metabolism, the next step is to replace purely geochemical catalysts with precursors of enzymes, perhaps oligopeptides and cofactors. A relevant observation is that dipeptide chirality can affect the chirality of the products of certain reactions (Pizzarello and Weber 2010); some dipeptides are better catalysts for particular reactions than others. Then, it is advantageous to increase the catalysts concentration. To this end some kind of memory device is needed. The development of protometabolism into metabolism must be inseparably entwined with the emergence of the genetic code.^18^ We expect peptide-polynucleotide coevolution (cf. Carter et al. 2014), but no experimentally testable scenario has been devised.

As Orgel (2008) pointed out quite eloquently, without enzymes there is no viable autocatalytic cycle.^19^ Thus, Szathmáry (2006) claims that the requisite degree of metabolic channeling is one of the biggest (if not the biggest) hurdles for the origin of life. However, if the fountain intensity is sufficiently strong, it is possible to maintain a protometabolic system around the fountain like a flame around a natural gas well. Perhaps, it is possible to make a “homotopy” between the full reliance on a fountain and a fully weaned system. That is, even not-so-efficient enzymes could sustain the autocatalytic systems if the help of the fountain is available.

Evolution is generally believed to require some sort of compartmentalization (privatization). Is there any way to make compartmentalization (cellularization) and use of the fountain of life compatible? A way is to regard a fountain as a compartment. That is, there is a possibility that initially, “cells” were not microscopic. Notice that a cell must be equipped with a reasonably complete metabolic system to sustain itself. Thus, for a microscopic cell to be possible, all the reactions must be microscopically regulated and compartmentalized. Without efficient enzymes this is quite unlikely. There must not have been any microscopic cells until protometabolism was complete to a certain extent.

Usually, cells are considered from the beginning microscopic, but different possibilities should be seriously considered. The simplest possibility is that the metabolic unit is a macroscopic system; if it is constructed around a fountain, it is possible that the system around the fountain may be a prototype of the cell. For a cell to wean from the fountain, it must contain a fairly complete metabolic system, which must be made compact. Such a compact system is possible only after considerable sophistication; some technological advancement is required for a cell to be small. Thus, the protocell system should not necessarily be microscopic and weaned from the fountain. This picture of “macroscopic protocell system” fits well with our nonequilibrium-driven scenario.

## Footnotes

1 Fry (2000) wrote, “*origin*-*of*-*life scientists*, *purposefully or inadvertently*, *implicitly or explicitly*, *by the very act of conducting their research are making a philosophical decision and taking a metaphysical stand*” (loc. 2649- of the Kindle version, stated in the context of criticizing religious influences).

2 FR is referred to as “fundamental conditions” by Oono (2012).

3 Cf. The semigroup word problem (Garey and Johnson 1979).

4 Of course, if a metabolic pathway is dissected in detail it often contains spontaneous fast reactions steps, but they are never “self-standing,” i.e., sandwiched between the narrow gates.

5 Few enzymes alter the chemical mechanisms of the reactions they catalyze. Thus, much can be learned about enzymatic mechanisms from the study of nonenzymatic model reactions (cf. Voet and Voet 2011).

6 The chemicals that do not react with the members of the existing network become part of the solvent or could be a catalyst or a cofactor.

7 One might say all the enzymes are complex molecules, but the enzymes mentioned here such as nickel containing ones (Ragsdale 2009) are especially large and complex enzymes.

8 Here, we may utilize a single chemostat that preserves the same (pre) biosoup as the initial state of the reactor. Just as in the case of thermostats, if we may use more than one baths, we could maintain the reactor under a nonequilibrium condition. To avoid nonequilibrium auxiliary conditions, we can use only one chemostat. The chemical reservoir, however, can contain as many chemicals as we wish, as long as it is in equilibrium.

9 The disequilibrium systems discussed in the conventional irreversible thermodynamics are systems with constant dissipation with (rather) steady irreversible flows that are driven by the free energy gradient. These irreversible processes explore the phase space while consuming the free energy. Cells, however, usually do not use the driven dissipative processes for the phase space exploration. For example, during the translation process in a ribosome, one must not forget when the free energy is consumed. Only when correct chemical combinations are formed, GTP is converted to GDP. That is, GTP is not used to search for good configurations, but to preserve good configurations that are formed by chance. Before this conversion occurs the system is virtually in equilibrium without dissipation. After the conversion, very quickly the system reaches another equilibrium state. The system, therefore, is almost “punctually” in equilibrium. In this case the state with GTP and that with GDP coexist without reaction for almost all time except for the occurrence of the correct configuration. Thus, such a system is said to be compartmentalized (in this case not spatially, but thermodynamically it is just as a compartmentalized equilibrium system consisting of subsystems in their own equilibrium states separated by walls) with constraints.

10 Here, the chemical network is considered in the context of the origin of life, so it is called a protometabolic system.

11 This may sound a convoluted way of saying a source of free energy, but we must emphasize that the free energy come with a particular compound or a few particular compounds. Thus, we must refer to fountains.

12 “Beneficial” implies, e.g., very efficient, very convenient, etc.

13 Examples of “high tech” reactions include the use of σ-complex of methane on rhodium and use of semiconductor nanoparticles or quantum dots to catalyze redox reactions (Eggins et al. 1998; Sadeghi and Nejat 2013; Tvrdy et al. 2011). However, “high tech” or “relict” is not an issue here. From the point of view of the common-sense prokaryotic biochemistry, too convenient reactions are called “high tech” and some of such reactions might perhaps be found as relict reactions.

14 If the logic to support many cofactors as the relicts of the RNA world is admissible, the argument here is certainly allowed.

15 Polymers might have been useful as we know even random polypeptides have some catalytic capability; they could even function as reserves of monomers. However, unless their ample supply is possible, their role as catalysts is secondary to more abundant catalysts that are geochemically supplied such as mineral surfaces and inorganic colloids.

16 There is no mitochondrion-less eukaryotes that did not lose mitochondria secondarily. Of course it is quite likely that we will not find any precursor of the present-day eukaryotes before the beginning of this endosymbiosis. However, still, there are lots of features we can infer about the predecessor (e.g., Shih and Matzke 2013). In any case, total discontinuity is quite unlikely, and erasing all the signatures of the preceding regime is highly unlikely.

17 If we assume CO_2_-rich atmosphere, photolysis of CO_2_ supply significant amount of CO continuously (Kharecha et al. 2005). An important sink of the atmospheric CO is reaction with H_2_O producing formate particularly under alkaline condition. This means that formate should be supplied continuously into the ocean under the CO_2_-rich early atmosphere. The formate may partly be converted into acetate (CH_3_COOH) via photochemical reaction within the surface ocean. This scenario should be tested experimentally (personal communication with Yuichiro Ueno).

18 What is the nature of this genetic code? It is clear that the code has nothing to do with polymers. Therefore, the code must have had some relations with chemistry or physical chemistry of amino acids. Thus, the authors tend to believe the original genetic code was actually the direct ancestor of the current genetic code as strongly suggested by the relation between codons and amino acid synthetic pathways (Taylor and Coates 1989; Copley et al. 2005).

19 As stressed by Eschenmoser (2007), there are not so many autocatalytic reaction examples. The important point Orgel (2008) emphasizes is that an autocatalytic cycle containing about 10 members cannot be effective as an autocatalytic system without excellent selectivity and yield (much better than 90%) of each reaction step. Thus, without enzymes no realistically significant autocatalytic cycle is possible.
